# Working Dog Training for the Twenty-First Century

**DOI:** 10.3389/fvets.2021.646022

**Published:** 2021-07-27

**Authors:** Nathaniel J. Hall, Angie M. Johnston, Emily E. Bray, Cynthia M. Otto, Evan L. MacLean, Monique A. R. Udell

**Affiliations:** ^1^Canine Olfaction Lab, Department of Animal and Food Science, Texas Tech University, Lubbock, TX, United States; ^2^Boston College Canine Cognition Center, Psychology and Neuroscience Department, Boston College, Chapel Hill, MA, United States; ^3^Arizona Canine Cognition Center, School of Anthropology, University of Arizona, Tucson, AZ, United States; ^4^Canine Companions for Independence, National Headquarters, Santa Rosa, CA, United States; ^5^Department of Clinical Sciences and Advanced Medicine, School of Veterinary Medicine, Penn Vet Working Dog Center, University of Pennsylvania, Philadelphia, PA, United States; ^6^Cognitive Science Program, University of Arizona, Tucson, AZ, United States; ^7^Department of Psychology, University of Arizona, Tucson, AZ, United States; ^8^College of Veterinary Medicine, University of Arizona, Tucson, AZ, United States; ^9^Human-Animal Interaction Lab, Department of Animal & Rangeland Sciences, Oregon State University, Corvallis, OR, United States

**Keywords:** training, conditioning, detection dogs, assistance dogs, behavior, learning, working dogs

## Abstract

Dogs are trained for a variety of working roles including assistance, protection, and detection work. Many canine working roles, in their modern iterations, were developed at the turn of the 20th century and training practices have since largely been passed down from trainer to trainer. In parallel, research in psychology has advanced our understanding of animal behavior, and specifically canine learning and cognition, over the last 20 years; however, this field has had little focus or practical impact on working dog training. The aims of this narrative review are to (1) orient the reader to key advances in animal behavior that we view as having important implications for working dog training, (2) highlight where such information is already implemented, and (3) indicate areas for future collaborative research bridging the gap between research and practice. Through a selective review of research on canine learning and behavior and training of working dogs, we hope to combine advances from scientists and practitioners to lead to better, more targeted, and functional research for working dogs.

## Introduction

Dogs have long been “co-workers,” collaborating with humans to complete a myriad of jobs in addition to providing companionship. Dogs have served, and currently serve, as shepherds, livestock guards, mobility assistants, therapy assistants, law enforcement canines, and supplement many more jobs. Some of the earliest reports of dogs in working roles involve assisting in hunting, dating back to at least 9,000 years ago (1, 2), as well as managing livestock and serving to some degree in wars, dating back to the times of the ancient Greeks (3, 4). Over the last 100 years, the practice of training working dogs and the science of animal behavior and training have both made significant advances. Traditionally, however, the practice and the science of animal training have developed in separate domains, with little “cross-talk” or collaborative efforts to advance both fields simultaneously. The objective of this narrative review is to briefly describe the history and scientific advances of the study of animal behavior that we view as applicable to working dog training practices, and then to identify areas for future collaborative research between researchers and practitioners to advance training practices for the twenty-first century.

Although the term “working dog” encompasses dogs that perform a wide range of functional activities, we have limited the scope of this review to three primary types of working dogs, which in present times reflect a majority of working dogs: protection/apprehension dogs, detection dogs, and assistance dogs.

### The Origins: A Brief History on the Origins of Working Dog Training

#### Protection/Apprehension Dogs

The use of working dogs for hunting, shepherding, and for some roles in war date back to at least classical Greek times (4, 5). However, formalized training manuals and procedures did not become more common until the 20th century (6, 7), although early treatises on dog training had long been available (e.g., Xenephon's Cynegeticus). In ancient Greece, dogs' role in war is subject to debate, as either trained participants or simply placed in war, but their current duties include sentinel, tracking, protection, and detection work (4, 8). Arguably, one of the earliest and most influential training manuals for the more modern use of protection dogs is Col. Konrad Most's “Training Dogs: A Manual” published in 1910 and reprinted in 2014 (7). This text has served as the basis for military and law enforcement dog training even until present day.

Although many of the training recommendations provided by Col. Most's manual use compulsion-based methods and are outdated (see section Reinforcers and Motivation), his impacts on the field are immeasurable. Col. Most and Oscar Pfungst [the researcher that identified the Clever Hans phenomenon: (9)] teamed together in the early 20th century to conduct, what remains today, one of the most robust evaluations (and debunking) of dogs' tracking capabilities (10). Col. Most was a practitioner-scientist, conducting extensive experimental tests on dogs' tracking abilities. By developing an intricate wheel to leave shoe imprints and zip line systems to move a human after laying a track, Most experimented to identify the signatures dogs use when following tracks and found that the human was not actually necessary and dogs will readily continue to follow tracks left by a wheel device in the absence of human odor (11). This finding led Most to suggest that dogs are tracking a complex stimulus “picture,” whereby the odor of damaged plants and visual stimuli also significantly contribute to the tracking performance of dogs. To a similar note, it is also worth noting that even earlier, Romanes (12) conducted an interesting series of small tests with his own dog, to elucidate that his dog successfully tracked the smell of his boots.

#### Detection Dogs

Training of detection dogs (narcotics and explosives) is a relatively more modern phenomenon with initial research dating back to World War II, but with wide-scale adoption occurring during the Vietnam War (3). One of the original manuals for training detection dogs was written during the Vietnam War era as part of a research program by the Southwest Research Institute in San Antonio, Texas (13). Interestingly, in contrast to more compulsion-based training procedures, this work was influenced by the developing field of Behaviorism (see section Early behaviorism) with significant influence from contemporary animal behavior research on positive reinforcement, reinforcement schedules, and minimization of aversive techniques. This manual highlights the use of schedules of reinforcement as well as controlling for and establishing food motivation.

#### Assistance Dogs

Assistance dog is an umbrella term that refers to a dog who is specially trained to provide support to a handler, enabling that person to live more independently by executing learned commands while also likely providing psychosocial benefits (14). Initially, most assistance dogs were guide dogs—i.e., dogs, matched with handlers who are blind, who aided in successful movement and navigation (14, 15). Over the past 45 years, the roles of assistance dogs have expanded considerably (16). For example, hearing dogs, matched with a handler who is deaf or hard of hearing, alert their handler to key sounds in the environment. Service dogs, typically matched with a person with a physical disability, perform tasks for this person that would otherwise be difficult or impossible for the person to perform (e.g., opening doors or picking up dropped items).

While depictions of dogs serving as guides for their blind handlers can be traced back centuries, the first official training school opened in Germany in 1916 (17). A little over a decade later, The Seeing Eye became the first guide dog school established in the United States. Thanks to the efforts of its initial head trainer, Eliot “Jack” Humphrey, its early breeding and training program also stood out for being extremely well-documented (18, 19). And in fact, many elements of the structure, timeline, and philosophy of the training pioneered by The Seeing Eye program remain characteristic of large schools in the guide and assistance dog industry to this day.

For example, given that so much of a guide's work transpires in public places, which always introduces a degree of unpredictability, early socialization to a wide range of environmental features is of the utmost importance. Therefore, early on, a “canine ‘Head Start’ program” was adopted, whereby volunteer families welcomed a puppy into their home and were responsible for the dog's early upbringing and basic obedience training [(17), p. 259]. This model is still commonly employed, with varying levels of supervision by the organization, until prospective assistance dogs reach adolescence e.g. (20, 21). It is then common for the dogs to return to a dedicated campus between 14 and 20 months, where they are housed in kennels as they progress through professional training. There is some converging evidence that the ideal age for the transition from puppy raiser home to professional training environment is around 17 months (22, 23). During professional training, dogs are taught the skills necessary for their future jobs, culminating in a multi-week joint training once matched with their new partner (17).

As part of his training philosophy, and in contrast to police dog training at the time, Humphrey was less interested in teaching compulsory obedience and was instead invested in allowing dogs to make their own decisions, even if that meant disobeying a command (17). In terms of training methods, this mindset translated into a lack of corporal punishment, as well as a greater emphasis on the dog and handler developing a rapport, facilitated through appropriate body language and vocal tones (19). Furthermore, Humphrey felt that it was the job of the trainer to adapt to a specific dog's preferences. Because The Seeing Eye's approach differed from the standard dogma of the time, they required their instructors to essentially start from scratch, completing an intensive apprenticeship course tailored to their program and methods of choice (17). This strategy has since become a hallmark of assistance dog organizations—for example, The Seeing Eye, Guide Dogs for the Blind, and Canine Companions for Independence all require their instructors to undergo 3–4 years of specialized training, regardless of prior experience (18).

### Changes Across the Twentieth Century in Animal Behavior

Like the formal documentation/manualization of animal training, the formal study of animal behavior is also relatively new. What is now known as Classical conditioning was formalized and published by Pavlov in the late nineteenth century (24), only 13 years before the publication of Col Konrad Most's original training manual, and many of the advancements in science and working dog training practice unfolded contemporaneously.

#### Early Behaviorism

The foundations for early behaviorism were laid by the research and work of Pavlov in the 1890s (25) and Watson in the 1920s (26). Pavlov's systematic work on learning was paramount to understanding how stimuli associated with important outcomes (such as acquiring food) can subsequently come to control and elicit a variety of behaviors when presented alone (see section How Dogs Learn). This initial work, and fundamental objections to the more introspective psychology prevalent in Europe (26) led to the early behaviorist movements which focused psychology on the study of behavior rather than introspections. This opened up a range of new interest and opportunities for animal behavior research within the realm of psychology.

Another pivotal moment was the scientific re-formulation of Thorndike's Law of Effect (26, 27) into operant/instrumental conditioning, which focuses on the consequences of behavior as a modifier of the future probability of that behavior (28). This led to significant output of research on the fundamental behavior of organisms (29) investigating the effects of various reinforcement schedules (30), how stimuli can come to control behavior, generalization, discrimination (31, 32) and much more beyond the scope of this paper (33–36). Due to the focus of understanding the underlying principles of behavior, this movement led to the systematic study of model organisms, leading to advances in animal behavior. The focus, however, was understanding behavior under controlled laboratory conditions where the effects of stimuli and reinforcement histories could be easily standardized and analyzed individually. As a result, much of the research used only a few model animal species such as rodents and pigeons (29, 37), which only rarely included the domestic dog [some examples (38, 39)].

#### Ethology

In parallel to the development of Behaviorism—which originated primarily in Russia and the United States—the field of ethology (the scientific study of animal behavior) took root in Western Europe. In contrast to Behaviorism, ethology emphasized the importance of studying animals in natural environments, and through an evolutionary lens. Early ethologists emphasized the study of relatively innate behaviors, focusing heavily on the concept of instinct and species-specific behavioral repertoires (40). Among the key contributions of ethologists were the development of what has come to be known as Tinbergen's four questions. This framework proposes that an integrative understanding of any aspect of animal behavior requires explanation at four complementary levels of analysis, including ontogeny, proximate mechanisms, phylogenetic history, and function/adaptive value (41). The influence of this field and its perspective within the working dog field can be readily seen in trainers' focus on a dogs' “innate drive,” or similar concepts such as “hunting drive” or “predatory drive” for detection dogs.

The field of cognitive ethology built on the basic principles of ethology, with emphasis on the study of animal minds in naturalistic contexts (42). Cognitive ethologists advocated for the use of field experiments, which they argued could more meaningfully probe the cognitive abilities of animals than relatively sterile laboratory tests of learning and memory (43). Cognitive ethology also brought consideration of animal consciousness to the forefront, including the challenges of understanding the minds of animals with radically different umwelts (i.e., the organism's experience of the world) than humans. Relatedly, Applied Animal Behavior (sometimes called applied ethology) emerged in the 1970s (44) in an effort to apply ethological concepts and behaviorism to issues in animal welfare and behavior. Applied animal behaviorists emphasize the importance of accommodating species-specific motivations and behavioral repertoires to improve animal welfare, often applying this knowledge to the management of captive populations. With respect to dogs, applied animal behaviorists often focus on remedying common problem behaviors such as aggression and compulsive behavior (45). Accounting for the role of the animal's immediate environment helps both in identifying the underlying cause of behavioral problems, as well as determining effective treatment and management of the behaviors moving forward. While applied animal behaviorists most commonly deal with pet populations, the same general principles can be applied to working dog populations.

#### Cognitive Sciences

The discipline of what is now known as cognitive science encompasses ideas and techniques from a number of related fields including psychology, computer science, philosophy, anthropology, linguistics, and neuroscience. Although many of the topics that cognitive scientists study were well-developed by the early 1900s, the formal origins of this field are often attributed to a period beginning in the 1950s termed the “cognitive revolution” (46). Thought leaders of the cognitive revolution rejected several key positions of early behaviorists, including the notion that the scientific study of psychology should be limited to observable behavior, or that associative learning alone could account for the majority of complex cognitive processes. Rather than treating the mind as a “black box,” cognitive scientists advocated for conceptualization of the mind as an information processor, analogous to those being developed by computer scientists. Like ethologists, cognitive scientists rejected the idea that minds were “blank slates,” programmed largely through reinforcement history, and instead emphasized questions about innateness, modularity of mind, and species-specific cognitive processes.

#### Veterinary Behavior

The field of veterinary behavior is relatively new with the first board certification exam taking place in 1995, although the interest and need for clinically-oriented behavioral work was recognized and started much earlier (see https://www.dacvb.org/page/History for a brief history). The focus of this field is to bridge the gap between medical knowledge and behavioral-health issues, as well as to advance the diagnosis and treatment of severe animal behavioral issues. This growing field continues to help attend to and treat the behavioral health of working dogs (47).

#### Comparative Psychology

The field of comparative psychology (sometimes referred to as animal cognition) captures the intersection of the fields described above and has been heavily influenced by work in each of these areas. Thus, rather than representing a distinct field, comparative psychology describes a broad domain of research concerned with the study of animal minds, which draws on concepts from behaviorism, cognitive science, ethology, and other related disciplines.

## Working Dog Training: Combining Research and Practice

### Canine Sensory Abilities

Before addressing how dogs learn, it is important to briefly address how dogs sense their world around them and explore the canine *umwelt*.

#### Visual

As a full synopsis of vision in dogs is beyond the scope of this article, we direct readers to more complete overviews of vision in dogs (48, 49). There are three main points that are particularly relevant for working dog training. First, many aspects of vision in dogs (e.g., depth perception and visual field of view, given eye placement) are highly influenced by breed (48). Second, compared to humans, dogs' visual acuity is relatively poor. Most dogs have ~20/75 vision, meaning that dogs can distinguish something from 20 feet away that a human with typical 20/20 vision could distinguish at 75 feet away (48). Third, in contrast to humans who have trichromatic color vision, dogs have dichromatic color vision, seeing mostly on the blue-yellow color spectrum (48, 50).

#### Auditory

Currently we know relatively little about dogs' auditory abilities (51). However, we do know that although dogs have similar hearing ranges to humans at low frequencies, their hearing range at high frequencies is much greater than that of humans [67–44,000 Hz for dogs, compared to 31–17,600 Hz for humans; (52)]. Moreover, dogs are able to discriminate between a large number of sounds, ranging from barks (53) to human commands (54).

#### Olfactory

Interestingly, despite the widespread use of canines for a variety of detection tasks, canine olfactory sensitivity is poorly documented in the scientific literature. Olfactory capabilities can be roughly categorized into discrimination capabilities (ability to resolve differences between molecules or complex odor mixtures) and detection (the minimum odorant concentration required to detect an odorant).

Few studies have thoroughly explored the discrimination capabilities of the dog and how that may compare to our own sensory capabilities. From a physiological perspective, dogs are thought to have ~1,000 different types of functional olfactory receptors (55) which is greater than the estimated 400 for humans (56) but less than the ~1,400 estimated for rodents (55). However, how these differences in functional olfactory receptor types translate to differences in olfactory perception is not quite clear due to the complex combinatorial code of olfaction (57). Some researchers suggest humans have sufficient capability to discriminate between a trillion different odors (58), and that human olfactory discrimination capabilities are not as poor as frequently assumed (56), so the perceptual implications of dogs' increased functional olfactory receptor repertoire on their discriminatory resolution remains to be determined.

Surprisingly, dogs' detection sensitivity limits have only been measured for a handful of odorants [For a review see (59)]. Even then, differences in measures between different authors, or even different dogs within the same study, can span several 1,000 fold or more [(60), See examples of detection threshold for amyl acetate (61, 62)]. These discrepancies make broad generalizations about the canine sense of smell difficult, especially in comparison to the human sense of smell, which can sometimes compare quite poorly or even exceed the detection sensitivity of the dog (56). Despite the open scientific uncertainty on how to make generalized conclusions on canine olfactory capabilities compared to our own, evidence suggests that for many odorants, dogs can have several-fold better detection limits than our own, making them helpful partners in odor detection (59, 62).

Altogether, while much research remains to better understand canine sensory systems, the current research suggests that dogs' visual acuity is poorer than our own and may be related to breed. On the other hand, dogs' auditory sensitivity encompasses a greater range than our own. Their olfactory capabilities can also well-exceed that of humans, but it may depend on the specific odorant and how the subject is tested and evaluated. These findings do suggest, however, that for training our working dogs, auditory and olfactory cues will likely be very salient stimuli, and dogs' perception in these domains frequently exceed our own capabilities.

### How Dogs Learn: Behavioral Principles

Dogs have been demonstrated to learn though three key mechanisms: Pavlovian conditioning, Operant conditioning, and Social learning. Here we provide a brief description of each type of learning and provide a very brief review of (1) key research on the learning mechanism and (2) areas where we believe research and practice can be better studied for working dogs in the future.

#### Pavlovian Conditioning

Pavlovian conditioning is one of the primary mechanisms by which all animals learn. Pavlovian conditioning is a learning phenomenon in which an association between two stimuli is developed. Initially, an originally neutral stimulus comes into association via contingency and/or contiguity with a biologically relevant stimulus [such as food, water, warmth, etc. (35, 36)]. Here, the neutral stimulus becomes a conditioned stimulus (CS) as it comes to predict the biologically relevant stimulus (unconditioned stimulus; US) and the animal learns to emit a response (conditioned response; CR) when the CS is presented. The organism learns to respond to the CS even when presented in the absence of the US. The “classical” example is when repeatedly presenting the sound of a metronome immediately prior to feeding canines, the metronome (CS) alone will come to produce an anticipatory salivation response (CR) in the dogs.

##### Review of the Research

Although Pavlovian conditioning is one of the earliest described phenomena in the psychology literature, it remains an active area of research. A thorough review of Pavlovian conditioning is outside the scope of this paper but is covered in a variety of learning texts (34–36).

Pavlovian conditioning has several key roles in the training process for working dogs. First, Pavlovian conditioning is an important process in developing secondary reinforcers, or reinforcers that are learned through association with primary/biological reinforcers, like clickers or other secondary rewards a handler may use (63). Additionally, Pavlovian conditioning is frequently leveraged when dogs show fearful responses (64, 65), which often must be addressed with working dogs, as they are likely to encounter a wide range of frightening stimuli in their working environment but must continue to perform. Lastly, Pavlovian conditioning is a key component in detection dog training. It is frequently referred to as “odor imprinting,” in which an odor is associated with a food or toy reward, although this differs from the imprinting referred to by ethologists as a specific type of learning in early life (66, 67).

Despite the importance of Pavlovian conditioning in several aspects of working dog training, relatively little research has been conducted. In the pet-dog field, several researchers have evaluated the efficacy of using a conditioned stimulus, such as a clicker during training. A recent systematic review indicates the clicker training is an effective method of training (63), but the results are less clear and often non-significant when making comparisons between training that only uses the primary reinforcer or both a conditioned and primary reinforcer (e.g., Clicker Training (68–70). However, it does appear that clicker training may lead to greater resistance to extinction (68).

The use of Pavlovian conditioning has also been evaluated for efficacy in facilitating odor detection training in dogs (71–73). Dogs that have had Pavlovian conditioning to a target odor prior to formal training learned the odor-detection task significantly faster than control dogs (72). With a follow-up within-subject design, dogs also learned to respond to a target odor to which it previously received Pavlovian conditioning faster compared to a control odor (72). Additionally, Pavlovian conditioning to odor has been shown to lead to lower detection limits for the target odor (74), and it leads to greater resistance to potential disruptors of performance such as pre-session feeding, odor distractors and extinction (73).

##### Translation of Research and Future Directions

Although Pavlovian conditioning is an important and critical component in odor-detection learning, it may not always be leveraged in an optimal way. Many times, in early scent detection training, a reinforcer is “paired” with a target odor to later be detected. This “pairing” or association is usually done by physically placing the two items in close proximity, such as within the same hiding box. The strength of Pavlovian conditioned response, however, is largely related to the informativeness of the CS and its relationship with the unconditioned stimulus (75). Simply pairing two items spatially does provide some spatial contiguity/similarity that can lead to associative learning, but this may not lead to the strongest behavioral response to the CS (75, 76). Further, when working with odors with low vapor pressures, which produce minimal odor availability, the potential for the target odor to be too low of salience to gain the animal's attention for conditioning should be considered in these preparations as well as the potential that other odors from the reinforcer might overshadow or block learning of the target odor [For a review see (77)]. As an alternative to spatial pairing, the reinforcer can be presented directly after the presentation of the target odor, providing temporal contiguity and contingency (if target odor is presented, then the reinforcer will follow). Interestingly, very little work has directly compared spatially pairing the target odor and reinforcer vs. presenting them in a more typical temporal preparation. In one study, dogs were trained to alert to anise extract as the target odor in a two-alternative forced choice task using two methods (78). In one method, accessible food reinforcers were available in a bin of pine shavings containing the target odor, and the comparison bin held inaccessible food but not the target odor. In the alternative method, one bin held target odor and the other did not, but the reinforcer (food) was not placed in either bin. If the dog made a response to the bin with the target odor, food was delivered immediately after (experimenter-delivered food). The results indicated that dogs learned faster with experimenter-delivered food (i.e., temporal pairing), suggesting that with the spatial food-pairing procedure may have led to food odor interference with the target odor.

Further, it could be useful to explore whether Pavlovian-conditioned reinforcers can help maintain working dog behavior when primary reinforcers, such as food and toys, are unavailable or impractical to provide, because some research suggests conditioned stimuli can improve resistance to extinction (68). Additionally, although Pavlovian conditioning and counterconditioning are popular procedures to help address canine fears in the dog training industry, almost no research has been conducted to evaluate how these procedures could be leveraged to help prevent fear or treat working dogs that may be disqualified for minor and specific fears (e.g., gunshot fears, fear of an escalator). Although these procedures are likely broadly used in the industry, the scientific literature is largely inadequate to describe the most efficient procedure or to even generally document effectiveness of such procedures in working dog settings. Investigating and documenting successful procedures could be a fruitful area of future research, and perhaps lead to fewer canine disqualifications for fear-associated problems that might be addressed through behavioral modification.

Overall, there is a large body of research that remains to be done, applying Pavlovian conditioning to working dog populations. Specifically, we suggest that more research is needed that explores the most efficient ways to conduct initial odor-learning for odor detection dogs, the usefulness of conditioned reinforcers to maintain working dog behavior when reinforcers are unavailable, and the use of Pavlovian conditioning/counterconditioning to address fears in working dogs that may otherwise lead to their disqualification.

#### Operant Conditioning

Operant conditioning, also frequently referred to as instrumental learning, refers to learning due to the consequences of a behavior and is highly conserved across animal taxa (28). Behavior “operates” on the environment, leading to changes that can feed-back to the organism to change the future probability of that behavior [increasing or decreasing (28)]. This can be broken down into reinforcement (probability of future behavior increasing) or punishment (probability of future behavior decreasing). Additionally, reinforcement and punishment can be further broken down into positive and negative, referring to whether the consequence is the addition (positive) or removal (negative) of a stimulus. However, there remains debate as to whether the latter distinction (positive vs. negative) is functional and whether it should be abandoned altogether (79–81).

A thorough review of operant conditioning and its application with dogs is beyond the scope here, but many texts in learning provide thorough coverage of operant conditioning research (34–36). The focus of this review is therefore restricted to applications and questions within operant conditioning that are of particular importance for working dog behavior and performance.

##### Review of the Research

*Discrimination Learning*. A substantial number of tasks that we ask of our working dogs are discriminated behaviors under the control of some stimulus. Discrimination learning simply refers to an animal engaging in a specific behavior in the presence of a specific stimulus (and not others). Detection work provides a clear example of discrimination learning, in which an “alert” behavior is required in the presence of certain odors, but not others. However, discrimination learning also applies to all service and assistance dog tasks in which a specific behavior is expected following specified commands or in the presence of certain stimuli (e.g., navigating sidewalks, traffic lights, and crowds).

Although discrimination learning encompasses a large number of tasks that we ask of our working dogs, the methods and procedures to produce discrimination learning are typically treated as an accessory question and rarely a central focus of research. Rather, questions on discrimination accuracy/capability for various target odors or cognitive tasks have been the primary focus (82–90).

*Training for Complex Discrimination Learning*. Animals are able to make very subtle and complex discriminations. Dogs have been trained on complex olfactory concepts such as discriminating odors from individuals with cancer from those without [(91), e.g., (92)] or from individuals with diseases such as COVID-19 from those without (93, 94). This ability is not very surprising given the complex visual discriminations that other species have been trained on, such as natural concepts of trees vs. no trees (32), man-made vs. non man-made (95), pathology images of cancer (96), and artistic painting styles (97). These examples in other species do highlight, however, the range of complex discriminations that working dogs could potentially learn through discrimination training.

Much less research, however, has evaluated how best to train these complex discriminations. In non-canine work, research has indicated that to develop complex discriminations and concepts, it is important to train the animal with a wide range of examples/exemplars of positive stimuli (target stimuli) and negative stimuli (non-target stimuli). Thus, the size of the “set” of training exemplars is related to the animal's ability to correctly discriminate a complex concept (98–100). The same seems to be true for detection dogs learning complex olfactory concepts such as accelerants (101) and home-made explosives (102, 103), in which large numbers of training trials and examples appear necessary. However, the size of the training set and number of odor examples necessary to produce an accurate discriminated concept has not been formally evaluated in dogs.

Limited work, however, has evaluated training techniques for dogs to produce accurate discrimination of complex odor mixtures. Fischer-Tenhagen et al. (104) evaluated two methods to train dogs to identify mixtures of herbs with a target (chamomile) from mixtures without. The authors trained the dogs to either the target stimulus alone (chamomile) or to mixtures with chamomile to mixtures without. Dogs trained using either method were able to successfully respond to novel herb mixtures containing the chamomile, but dogs trained with the mixture procedure made more correct indications during the test phase. This study is similar to (102) that trained dogs to alert to ammonium nitrate and hydrogen peroxide odor mixtures. Dogs that were trained to the pure target performed poorer in generalization tests than dogs trained with odor mixtures with the target from odor mixtures without the target. Similarly, Lazarowski and Dorman (103) demonstrated that dogs struggled generalizing from detection of pure potassium chlorate to potassium chlorate mixtures unless they received training with the potassium chlorate mixtures, further highlighting how training methodology could be an important variable influencing performance.

*Errorless Learning*. One potential method to facilitate complex and challenging discriminations for working dogs is errorless learning (105, 106). Under typical discrimination training procedures, the animal is free to respond to both correct and incorrect stimuli to learn that responses to correct stimuli are reinforced and responses to incorrect stimuli are not. In errorless discrimination learning, the relative salience of the correct and incorrect stimuli is manipulated at the start, such that the probability of a response to the incorrect stimulus is highly unlikely. The “incorrect” stimuli are then slowly faded in to reach a similar intensity/salience as the correct stimulus but done so at a rate that ensures the animal makes very few, if any, incorrect responses. The animal therefore learns the discrimination without making incorrect responses and is “errorless.” These procedures have demonstrated robust discrimination learning in pigeons (105–107); however, the scientific literature is largely lacking examples of applying this procedure for working dogs [note see one example (93)].

##### Translation of Research and Future Directions

Despite the widespread use of discrimination learning by trainers for working dogs, little research has focused on how it can be optimized for efficiency in training and performance. For example, an area that holds promise for future research is the application of errorless learning for odor detection dogs. In biomedical detection programs, dogs must discriminate from complex biological samples with an undefined odor for “disease” from highly overlapping complex odor samples from patients without disease. During initial training, this task can be quite challenging for a dog; however, errorless learning procedures can be conducted by manipulating the non-target stimuli. First, dogs can be trained to respond to target odors from blank (empty) comparison samples. The similarity of the incorrect samples to the target sample can slowly be increased, by presenting diluents, until reaching comparison samples that are otherwise identical to target samples. By following this arrangement, the dog is always likely to be successful, which can help maintain motivation for the task. Importantly, however, research comparing such a procedure to a procedure in which the dog must learn through trial-and-error, and the implications each training style has for detection sensitivity and specificity, has not been conducted. Such research represents an important future direction.

Another consideration for discrimination learning is to evaluate the optimal schedules of reinforcement for training working dog tasks. During initial acquisition, continuous schedules of reinforcement are frequently used. However, for maintenance, intermittent schedules could be leveraged. Intermittent schedules are reinforcement schedules in which correct responses are reinforced following only some of the responses and could follow a variety of different schedule types (108). The benefits of intermittent schedules of reinforcement are that they lead to greater persistence in behavior if an animal cannot always be reinforced [Partial Reinforcement Effect (109–112)]. Additionally, behavior appears most resistant to disruption and extinction when high rates of reinforcement are used compared to lower rates [(113–115), For a review see (116, 117)]. Together, these results suggest that applied parametric studies that evaluate different schedules of reinforcement for working dogs engaging in their relevant task could be a useful future direction to produce the most robust behaviors in distracting environments.

Although operant conditioning is a key process by which working dogs are trained, little research has focused on how to optimize training parameters to produce the most robust behaviors in real-world and distracting environments. Such research could be fruitful translational research to optimize training programs and enhance performance. Given the basic research on procedures such as errorless discrimination, concept formation training, and schedules of reinforcement, there are several potential training methods that could be deployed and evaluated for effectiveness and efficiency in training working dogs.

#### Social Learning

##### Review of the Research

Rather than representing a single learning mechanism, social learning refers to a constellation of learning processes in which information from other social agents influences the learning process. These processes range from simple cases of “enhancement” in which an individual's attention is directed toward important stimuli or locations via other agents, to imitative capacities, in which an individual acquires novel skills by observing and modeling the actions of others. As a highly social species, various forms of social learning are likely to be important to dogs. In the last two decades, much research on dog cognition has focused specifically on processes related to social cognition, yet little of this work has been integrated into applied training protocols. Below we highlight two promising areas of research, the first exploring conspecific social learning, and the second capitalizing on dog's abilities to learn socially from humans.

*Conspecific Social Learning*. Altricial species (in which newborn animals are relatively immobile and highly dependent on others for survival) generally have prolonged periods of intensive contact with parents. These periods present rich opportunities for social learning and tend to coincide with key stages of brain development (118). In wolves, pups begin to accompany adults to kill sites by around 10 weeks of age yet remain highly dependent on their parents and reside in the natal pack for at least the first 10 months of life (119). It is likely that social learning, particularly from parents, plays an important role during this period. Because domestic dogs are typically provisioned by humans and are often separated from their mothers by 8 weeks of age, this may eliminate important opportunities for social learning from adult conspecifics.

To explore the potential applications of conspecific social learning in working dogs, Slabbert and Rasa (120) conducted a study in which German shepherd pups were separated from their mothers at either 6 or 12 weeks of age. The latter group was allowed to observe the dam performing detection work between 6 and 12 weeks, presenting an opportunity for social learning. Compared to pups separated from their mother at 6 weeks of age, pups who observed the dam performing narcotics detection from 6 to 12 weeks scored significantly higher when trained and tested on this task at 6 months of age. Although the specific mechanisms of social learning were not identified, the finding suggests that social learning occurred spontaneously (dogs were not trained to attend to or mimic the actions of the dam) and facilitated subsequent training as a working dog.

*Social Learning From Humans*. Dogs readily learn from humans (121, 122). For instance, dogs follow human pointing from an early age [e.g., (123, 124)], and even imitate human actions in some contexts (125–127). Research investigating the “Do as I Do” method is particularly relevant for considering social learning in a training context. In this method, dogs are trained to imitate the same action that a human performs upon hearing the “Do it!” command (125–127). After a period of substantial training, dogs can learn to readily imitate human actions following the “Do it!” command, even after a 24-h delay (127).

In fact, not only do dogs readily learn from humans, but they communicate with humans as well. In particular, when dogs encounter an unsolvable (128), challenging (129), or fear-provoking task (130), they “look back” to humans and make eye contact. Importantly, though, there are differences in dogs' tendency to look back based on their training background (131–133). Most notably, highly trained dogs are less likely to look back to humans than less trained dogs (131, 133). This research highlights two important features of social learning in dogs: (1) dogs are ready to learn from humans from an early age and (2) experience, especially training experience, can influence how dogs engage with humans in a social learning context.

In fact, pet and working dogs sometimes follow human social cues even when it is not the most beneficial solution to a problem. For example, in addition to looking back to humans when confronted with an unsolvable task, dogs (pet, shelter, and free-roaming) spend almost as much time looking back at a human handler standing neutrally nearby when confronted with a novel solvable task (129, 134, 135). Likewise, studies have also demonstrated that dogs will sometimes choose to follow human gestures to an empty container, even if they can see and smell that food is located in an alternate one, and compared to non-domesticated canids, dogs persist at gazing toward humans who previously provided food or attention for much longer after human attention/responsiveness has been withdrawn (135, 136). Recently, research has identified several key genetic differences between dogs and wolves, including structural variants in the GTF2I and GTF2IRD1 genes that are associated with a hyper-social predisposition in dogs and correspond with heightened social focus on these tasks. However, genetic variation in this region has also been identified between dogs (137), and there is ample evidence that lifetime experience plays an important role in the social development of dogs (138). Therefore, it is not surprising that when dogs are given verbal and gestural encouragement to focus on an independent solution to a task, their persistence rates significantly increase and they look back to the human less frequently (129). Trained working dogs are more successful at translating this increased persistence into task success (129, 135), demonstrating that training style and history heavily influence the impact of human presence on the dog's behavior, focus, and task outcomes.

#### Translation of Research and Future Directions

The above research highlights how dogs are prepared to learn from social partners, but also highlights possible challenges that could occur during training or when employed in a working role. Critically, simply removing the human from the environment may not be the answer for animals used to working as part of a team, as the absence of the human partner can also lead to abnormal performance behaviors and decreased persistence (129). Especially in working roles that require the dog to engaged in independent action, the ease with which dogs may be unintentionally influenced by the actions and subtle cues of a handler or others in the environment should be considered. Prior work has shown that detection dogs can be sensitive to subtle cues and that a handler's belief about the presence of an odor can lead dogs to higher rates of false alerts (139). Additionally, more recent work has shown that handler knowledge about the number of target odors hidden can influence the length of the search and the frequency at which the dog looks back to the handler, but did not ultimately lead to differences in false alerts (140). These results suggest dogs' sensitivity to human action can be both a help and a hindrance; however, greater awareness of human influence can help shape practices that better control for unwanted influence and utilize dog's acute awareness of social stimuli to the working team's advantage.

To our knowledge, the explicit application of social learning in working dog programs remains relatively rare. However, we propose several ways in which research on social learning may pertain to working dog training programs. With respect to conspecific social learning, working dogs are commonly separated from their litters by 8 weeks of age, limiting opportunities for social learning that might be more common in the socioecology of feral dogs or their evolutionary forebears. Thus, waiting to separate dog pups from their mother and allowing dog pups to observe their mother performing tasks until 12 weeks of age might lead to enhanced training outcomes. Alternatively, if delaying transition to foster homes leads to fewer exposures and experiences outside of the whelping and kennel environment for the puppies, this recommendation may be contraindicated and lead to more fear, highlighting the need for more research to establish best practices.

In addition to this early-life social learning, there are other important opportunities for conspecific social learning that can be fostered (and experimentally evaluated) in adult working dogs. For example, many working dogs are trained at dedicated facilities in which individual dogs alternate between bouts of active training with a human handler, and periods of rest and downtime while the trainer works with other dogs. These periods of downtime, however, may present opportunities for social learning, especially if dogs have opportunities to observe other dogs actively being trained. The potential utility of this approach could easily be evaluated using experimental designs in which some dogs are given rest and downtime in isolation whereas others are given opportunities to observe other dogs in training during these periods.

Assistance dogs, detection dogs and search-and-rescue dogs frequently need to be habituated to potentially fear-provoking scenarios such as navigating large crowds, navigating escalators, walking over rubble or being exposed to gunfire. Frequently, dogs are habituated or desensitized to these conditions individually. Social learning literature suggests there may be benefits to a dog first observing a conspecific navigating these scenarios comfortably and confidently. However, such experimental research has not yet been done, and so would be interesting and useful to conduct in the future.

Additionally, with respect to social learning from humans, dogs can be trained to imitate human actions using the Do-as-I-Do training program. This training program allows more flexibility in the types of actions that dogs can learn to perform as they simply imitate whatever action the human performs and can retain this information even after a considerable delay. Notably, incorporating Do-as-I-Do involves a substantial initial investment in training the “imitate” command, but may pay dividends if it can be subsequently used to rapidly train a variety of other behaviors. Thus, we expect this approach may be particularly useful for dogs required to master large repertoires of trained behaviors (e.g., service dogs) more so than dogs who are trained to perform a smaller set of commands. Notably, once established, Do-as-I-Do training has been shown to facilitate faster skill acquisition than traditional operant techniques, and is associated with more robust transfer of trained behaviors to novel contexts (126).

#### Reinforcers and Motivation

##### Review of the Research

A combination of evolutionary, genetic, developmental, and lifetime factors may influence the salience of specific stimuli to an individual, breed group or to dogs in general. This is true for both perception and responsiveness to stimuli that precede a behavior (such as releasers or discriminative stimuli) and stimuli that follow/act as a consequence for the behavior, including re-inforcers and punishers. For example, breeds of dogs traditionally selected for a strong motivation to chase (e.g., Border Collies or German Shepherd dogs) may be predisposed toward greater responsiveness to a variety of moving stimuli compared with dogs from breeds selected for inhibition of these traits, or a higher response threshold to moving stimuli, such as Anatolian Shepherd dogs and Great Pyrenees (141). Genetic differences associated with attentional bias toward social stimuli have similarly been found to correspond with assistance dog success (137). When the working role of a dog requires behaviors that are associated with motor patterns that have biological relevance, or motor patterns that have been selected for within a specific working breed, the motivation for engaging in the behavior may be intrinsic and require less shaping and external reinforcement than when dogs are being trained to display behaviors or do jobs that are less related to their natural behavioral repertoire. Therefore considering the domestic dogs behavioral ecology, as well as motor patterns and biological predispositions under selection when breeding working dogs, may inform what jobs dogs will do best, and inform training practices in ways that allow handlers to utilize a dog's predispositions and motivations to aid the training process (142).

Socialization and lifetime experience are also known to greatly influence how dogs perceive and interact with stimuli in their environment and can also contribute to motivational factors (138). Other motivational factors, including what establishing operations (i.e., environmental circumstances that make a behavior more or less likely) may best set the stage for effective training or job performance, are also important to consider. For example, factors such as the timing and duration of the training session, degree of hunger or thirst, temperature, time since last entering the training area, or interacting with the trainer, and many other factors can influence motivational state and therefore a dog's inclination to focus and persist on training tasks. Furthermore, considering how environment and motivational factors may differ between a training environment and final work setting can be used to simulate final working conditions or highlight the importance of training sessions in applied settings. Likewise, not all dogs (even within the same breed or training program) will find the same items or activities as reinforcing as others (143). In fact, what some dogs find reinforcing, others may find aversive, or frightening (144). Additionally, the same dog may not find the same items or activities reinforcing or aversive all of the time.

Dogs may also differ in degree of persistence Rao et al. (145), inhibition (129, 146), or baseline arousal levels (147), which in turn may influence what reinforcement schedule is optimal. Because of this variability, it is not possible to describe the ideal motivational considerations and reinforcers for all working dogs here, although there is a great need for more research looking at the efficacy of training practices, including managing establishing operations and reinforcers, across a wide range of working settings. However, several concepts well-studied in the literature across a broad range of species (including dogs) may serve as a scientific basis for deciding what motivational and reinforcement strategies could work best for individuals or groups of dogs within a specific training context.

##### Translation of Research and Future Directions

*Preference Assessments*. Identifying the high-valued reinforcers is critical for a successful training program. Frequently, dogs are selected based on whether a certain reinforcer is highly motivating for a dog (i.e., “ball drive” or “toy drive”) through a variety of selection tests (148). An alternative concept that may help prevent the failure rate of dog training programs is to provide preference assessments that allow the trainer to select highly motivating reinforcers for the dog, rather than selecting a dog for the reinforcer. For example, potential working dogs could be evaluated for motivation for a variety of food, social, and toy rewards to create a hierarchy of reinforcers that could be used. The methods of preference assessments themselves are generally well-established (143, 149–151), making this strategy straightforward to implement. Additionally, by establishing a range of potential reinforcers, issues associated with satiating one reinforcer may be prevented by having alternatively available reinforcers. Further, this strategy would also allow for a range of studies investigating whether reinforcement schedules that provide varying reinforcer types may lead to more persistent behavior less susceptible to satiation.

*Establishing Operations*. Maintaining and controlling reinforcer value is important to preserve any trained behavior. However, how best to do so for working dogs has not been previously researched and would be a useful future direction. Various agencies have practices to help establish their reinforcers as high value, such as only allowing the dog access to the reinforcer during training, whether it be a certain type of toy or food. However, it is not clear whether these more extreme schedules are necessary to establish the desired behavior. It is possible that selecting dogs with a “high drive” for a particular reinforcer and only providing access through irregular training may lead to the development of alternative or undesired behaviors. For example, high levels of motivation produced through deprivation can lead to higher levels of generalization (152), which could produce new behaviors or responses to non-target odors that are undesirable. Further, in many working dog applications, much emphasis is placed on selecting dogs with extreme motivations for reinforcers such as toys. It is unclear, however, whether ultimate performance as a working dog is linearly related to reinforcer motivation (i.e., more “drive” leads to better performance). Alternatively, there may be an inverted-U function for some tasks that require attentiveness, in which there is an optimal level of motivation for the reinforcer and very low or very high levels of motivation may each produce performance decrements. Thus, future research manipulating establishing operations and its impact on working dog performance may be a useful future direction to optimize consistent performance and motivation.

*Use of Aversives*. Incidental effects from the use of aversives have been documented in the basic research literature, such as elicited conspecific aggression (153), fear of punishment associated stimuli (154), and substantial suppression of all behavior within a punishment context (39, 154, 155). Growing applied literature with dogs highlights that positive reinforcement based training is effective and the use of aversives can have negative welfare side effects for the dog (156–161). This highlights the need to further consider not only how to motivate working dog behavior (e.g., does the dog engage in the behavior to receive a reward or to avoid a correction), but also which methods produce the best performance *and* welfare outcomes for working dogs. In this respect, treating our working dogs as “student learners” and evaluating how to arrange environmental conditions that set working dogs up for success may promote successful performance and welfare outcomes.

#### How Dogs Think Can Inform our Training

##### Review of the Research

A full overview of research on how dogs think is outside the scope of this article. For a more comprehensive overview see Bensky et al. (162). At the broadest level, there is a growing body of work on how dogs think about both the social and physical world. We cover each of these domains of canine cognition in the sections that follow.

*Social Cognition: Thinking About the (Human) Social World*. Given that the majority of research on social cognition in dogs has explored how dogs think about humans, we will limit this review to what dogs think about the human social world. These findings are of relevance to working dogs because they highlight ways in which working dogs may work with, learn from, and understand their trainers.

Perhaps most notably, dogs have some aspects of a “Theory of Mind” and are able to make inferences about some human mental states (163). In particular, dogs are able to interpret a human's visual perspective [i.e., understand what a human can see (164, 165)] and they also seem to expect that humans will remember what they have seen [i.e., have knowledge of what they have seen (166–168)]. Moreover, dogs respond to human intentions and can identify when a human intends to communicate with them (169) and when humans are performing goal-directed actions (170). In addition to evaluating human mental states, dogs respond to human emotional states (130, 171, 172). For instance, dogs will fetch an object that a human has emoted positively toward in the past (172) and are more likely to go toward a scary object if their owner has emoted positively toward it (130). Further, some work suggests that training may impact a dog's tendency to react to a person's emotional state. In particular, in one study, dogs trained for water rescue were less likely than pet dogs to approach a novel object simply because a person had emoted positively toward it (173).

Building on dogs' understanding of human emotional states, dogs, in some cases (but not all), will help humans when they are in emotional distress and in need of help (174–177). Interestingly, work so far suggests that this tendency to help is not influenced by therapy training, as therapy dogs in one study were no more likely to help than non-therapy dogs (176), though more work is needed to explore the influence of other types of training. Furthermore, dogs will not only help humans in times of distress, but they will also cooperate with humans on joint goals (178). Interestingly, though, dogs struggle to spontaneously cooperate with one another (179).

In addition to understanding something about human mental states and emotions, there is evidence that dogs can evaluate humans based on who has recently been “nice” or “mean” in some contexts (146, 180–182). However, this ability may be limited only to those dogs who receive certain types of training. One recent study suggests that only agility trained dogs, not pet dogs, showed a preference for a helpful experimenter over a hindering experimenter (183).

Finally, there is growing evidence that dogs have some understanding of human language (184, 185). Not only are dogs able to learn the names for many objects, they can learn new words via a system of “fast mapping” wherein they learn the names of new objects via a process of exclusion (184). Specifically, if they know the name of three objects, and someone requests a novel word (e.g., “blicket”), dogs are capable of inferring that the fourth object they do not know the name of must be “blicket.” Building on this, there is some evidence that dogs are capable of understanding language syntax (185). However, it should be noted that this work has been done with a handful of highly trained dogs, so it is unclear to what extent these findings generalize to other dogs.

Thus, in at least some cases, dogs understand human intent and emotions, provide help to humans when they are emotionally distressed, cooperate with humans, prefer humans who are “nice” over those who are “mean,” and show some comprehension of human language. Taken together, these findings have the potential to impact working dog training because they highlight ways in which working dogs may relate to their trainers, understand their trainers' behavior, and language.

*Non-social Cognition: Thinking About the Physical World*. Understanding how dogs think about the physical world is crucial for supporting best practices in working dog training because it gives us insight into the ways dogs think about and see the world. Through understanding dogs' cognition, we can determine which training practices interface best with their understanding of the physical world.

Dogs understand many of the same features of the world that human infants do. For instance, they have a basic understanding of object solidity [i.e., that objects are solid and other objects cannot pass through them (186)] and object permanence [i.e., that when objects are out of view they continue to exist; e.g., (187)].

Building on this, dogs seem to have at least a basic sense of number and quantity. They can distinguish between large quantities (e.g., 10 pieces of food or greater) and small quantities [e.g., 5 pieces of food or fewer (188, 189)], and in some cases can discriminate between small numbers under 5 [e.g., tell the difference between 1 and 2 (190)]. However, dogs' ability to discern objects based on number may be context-specific because they do not demonstrate this ability in every experimental context (191).

Another crucial aspect of non-social cognition is memory. Dogs seem to have a working memory capacity of a few minutes. Research has shown they are able to keep the location of hidden objects in working memory for up to 4 min (192). However, the duration of dogs' working memory, and other executive functions, decline with age (193, 194). In addition to working memory, some recent work suggests that dogs may be capable of the elements of episodic memory (195), including the what, when, and where of odor cues (196). Further, dogs' working memory for odors can be quite expansive, with recent research indicating dogs' odor working memory in an odor span task is upwards of 72 odors, which is similar to rats (197, 198). In terms of long-term odor-memory, dogs are able to readily learn to detect 10 different target odors successfully, but the experiment did not evaluate beyond 10 odors (199). Interestingly, little work has evaluated retention of odor memory in dogs, but a small study of three dogs found dogs maintained accurate odor discrimination performance after a 69 day delay (200). Most recently, extending these results Lazarowski et al. (201) found dogs' memory for odor recognition to remain largely robust over 12 months with minimal training. These parameters are critical for further exploration given that typical odor detection dogs are frequently trained to more than 10 target odorants, and it's unclear the necessary interval for “refresher” training to maintain optimal performance. Dogs are frequently given weekly training, but this may be unnecessary given the results of Lubow et al. (199) and Lazarowski et al. (201) but more extensive work is necessary before best practices can be established.

Recently, researchers have begun exploring the contextual factors that affect dogs' ability to remember learned tasks. Preliminary research suggests that engaging a dog in activities that likely induce “pleasant arousal,” such as walking and play, directly after learning a new task has positive effects on their memory for that task when tested again 24 h (202), 1 week (203), or even up to 1 year (204) later. In contrast, having them immediately engage in learning of an unrelated task results in cognitive interference, thereby disrupting memory consolidation (203). Interference in memory tasks also seems to be critical for odor memory in dogs (205). Sleep appears to be another crucial variable (206). In dogs specifically, performance in learning new commands has been shown to be enhanced by sleep-related improvement in memory consolidation (203, 207, 208). Given that command learning is an integral part of working dog training, these findings, along with an emerging literature on the environmental factors that affect quality and quantity of sleep (209), are of great relevance. For example, these findings can inform how trainers structure the duration and timing of their training sessions with regards to other activities, especially when teaching new commands. Perhaps shorter sessions over multiple days, separated by more opportunities for play and sleep, would pay greater dividends than packing multiple sessions into a single day.

One domain of non-social cognition where pet dogs do not excel is independent problem-solving. Compared to wolves and dingoes, pet dogs often struggle to figure out how to solve puzzles based on causal reasoning (210–212), and when dogs do figure out how to solve problems at the group level, there is often significant variation at the individual level (213). Given that pet dogs are less adept at solving physical problems than non-domesticated canids and demonstrate large individual variation in problem-solving abilities, some scholars have suggested that artificial selection may have relaxed selection pressures for independent problem solving (210). That said, training appears to impact dogs' propensity to solve problems, as highly trained dogs are more adept at solving physical problems than less trained dogs (131, 133). It remains unclear, however, whether this is a byproduct of training that enhances problem solving or rather reflects that dogs with enhanced physical problem solving are more receptive to advanced training.

Overall, when it comes to dogs' understanding of the non-social world, they have a basic understanding of object properties, some understanding of quantity and number, and a working memory capacity of a few minutes. That said, dogs are not naturally adept at individual problem solving, though training seems to enhance their ability. Taken together, these findings have the potential to impact working dog training because they highlight both strengths and weaknesses of dogs' cognition that may impact which training methods are most tractable.

##### Translation of Research and Future Directions

Although a growing body of work investigates the impact training can have on canine cognition [e.g., (131, 133, 173, 183)], no empirical work to our knowledge has yet integrated basic research on canine cognition with research exploring the effectiveness of various training methods. It is our hope that the brief review of canine cognition above will stimulate ideas regarding how to translate this basic research into effective training methods. However, a few findings in canine cognition are of note for trainers. First, it may be useful to keep in mind the areas in which dogs excel. Dogs track human emotional states [e.g., happiness, anger, disgust; e.g., (130, 171, 172)], and in some cases even their mental states [e.g., knowledge, goals; e.g., (167, 170, 214)]. This ability suggests that training involving these social cues may be effective. Additionally, research evaluating how sleep or engaging in enjoyable activities following training may facilitate memory and later performance could be of particular importance to enhance training success.

Likewise, it is important to keep dogs' cognitive limitations in mind in a training context. Notably, in the absence of formal training, dogs do not naturally excel at independent problem solving [e.g., (210–212)]. Thus, training methods that rely on dogs' individual problem-solving skills may prove less effective than other training methods. Moreover, although dogs do have a sense of number, this can be context-dependent and dogs do not demonstrate this understanding in all contexts e.g. (190), indicating that planning training situations that require dogs to make these discriminations would not be as effective or efficient.

#### Human-Animal Bond

##### Review of the Research

While there are multiple factors that may be important when considering how the human-dog relationship can influence working dog performance, there is growing evidence that the success and well-being of both the human and dog involved in a working partnership can be significantly impacted by the quality of bond shared between the two. Even in adulthood, dogs have been shown to form attachment bonds with humans that resemble conspecific infant-caregiver attachment relationships (215). While dogs can form bonds with new humans quickly (216), the quality of these relationships can vary, with some environments and experiences resulting in higher rates of secure attachment bonds than others (216–218).

Secure attachment refers to a persisting relationship between two individuals (in this case a dog and a human owner, handler, or trainer) that promotes proximity seeking, contact exploration balance, and stress reduction in unfamiliar environments or situations (219). While attachment is the product of a mutual bidirectional relationship, it can be assessed from both the perspective of the human (through behavioral evaluation or surveys) and the dog (through behavioral evaluation).

While the recognition that dogs and humans can share deep bonds is not new, research investigating the quality of these bonds from the dog's perspective has been limited. Only recently have scientists begun to ask about different styles of attachment that dogs show toward their caretakers, or the impact of attachment security (or insecurity) on the dog's behavior and welfare (217, 220, 221). While even less research has been done specifically on working dog attachment relationships, the quality of owner/handler bonds may be an important factor in training and job success. In fact, one study found that working search-and-rescue dogs were more likely to have secure attachments to their human partner when compared to pet dogs [although this difference was not statistically significant (219)]. If further research finds such trends are representative of a true population difference, it will be important to understand why. For example, an enhanced bond could be due to the influence of working dog training, or simply additional time spent with the human, on attachment quality, or conversely it could suggest that dogs with secure attachments are more likely to be successful working dogs. Additionally, secure attachments are, by definition, relationships that reduce stress, especially in novel, or unfamiliar contexts (222). Focusing on attachment or other aspects of relationship quality between dogs and their trainer(s), owner(s), and handler(s) may therefore be valuable in terms of sustainable and humane practices independent of whether the bond improves other aspects of training success (221, 223).

##### Translational Research and Future Directions

For many working dogs, training and/or work will require living in multiple places, as well as frequently working in new environments and/or with new people (224). While some trainers, fosters, or other short-term caretakers express concerns about developing a strong bond with dogs in their care temporarily- often for fear that breaking the bond will harm the dog when they are rehomed (225)- research to date suggests that forming a secure bond may instead have an important and positive impact on the dog's success and well-being in both that environment and the next. The broader literature has demonstrated that even when dogs transfer handlers, high quality secure attachments developed earlier in an individual's life can be beneficial and predictive of the formation of new secure attachments at later stages of life for both humans (226, 227) and dogs (223). Considering the other side of the relationship, evidence to date indicates that dogs with secure attachments to their human caregiver are on average more persistent, more exploratory and also show fewer behavioral signs of stress and neuroticism in novel environments (217, 220, 228). The quality of attachment that humans report sharing with their own dog, or a working dog partner, has also been found to be predictive of therapeutic benefits and/or the beneficial impact of that dog on the human's quality of life (218, 229, 230).

While a relatively new area of study, there is already some evidence that the attachment style of working dogs toward their caretaker or handler may be an important aspect of training success and job performance. Differences in focus or comfort level in novel situations, associated with the presence or absence of a secure base effect, could potentially impact training, or performance (224). For example, one study found that trained therapy dogs were able to perform the function of remaining near a mock therapy participant equally well, independent of their attachment style toward their handler. However, dogs with insecure attachments spent more time looking back at their handler (and away from the therapy participant) during the session (224). Such behavioral differences could be meaningful, as looking away may signal discomfort on the part of the dog and could also be interpreted as disinterest by the human therapy participant—which could reduce therapeutic success. However, more research is needed to better understand the impact of such outcomes. Dogs with secure attachments to human caretakers/handlers have also been found to show greater task persistence (217) and increased readiness to engage in object manipulation (228), traits often relevant to working dog training success. Given that, in humans, attachment style has been used to predict a wide range of factors related to executive functioning (231), learning success (232), and career success (233), more research into the ways that dog-human attachment relationships may impact a working dog's training and performance is needed.

## Formalizing Handler Expertise

In this review, we have identified several directions and needs for formal research with working dogs, thereby providing an important basis to move research in new directions. However, it is also critical to note the vast body of animal behavior knowledge that expert trainers have developed through daily experience. Much of this knowledge is unpublished, not widely available, and rarely appears in the scientific literature, even though it may have been developed over decades of informal testing. To fully move the field forward in a collaborative way, systematic research through expert interview and qualitative research methods could be a highly beneficial practice to formalize some of the procedures, thought processes, and experience developed by expert working dog trainers e.g. (234). Although expert trainers may not have a scientific explanation or justification for their observations or procedures, finding a way to formalize their years of hands-on experience and feedback (via dog performance) could yield a wealth of information that would save researchers valuable time and resources. It is important that practices are ultimately held up to an empirical evaluation, so formally documenting the lessons learned by expert trainers would be an important step forward to generate collaborative work that builds upon trainer expertise (rather than re-developing it), addressing the most critical questions important to both the researcher and trainer.

## Concluding Remarks

Working dogs are trained to complete a myriad of tasks for service, assistance, detection, and protection work with much success. And yet not all working dogs that enter training programs are successful (235), leading to high costs and limited availability of working dogs. Optimizing training efficiency represents one way to increase the probability that a dog will be successful. Over the last 100 years, our scientific understanding of animal behavior has grown and expanded rapidly and so has the expertise and methods of training working dogs. It is our hope that this review inspires new directions of collaborative research between researchers and working dog practitioners with the goal of expanding evidence-based information and techniques that future working dog trainers can incorporate. The synergy of these two areas will likely result in improved training practices and have a measurable impact on the outcomes, welfare, and availability of working dogs filling needed roles in our society.

## Author Contributions

All authors contributed to conceptualization, investigation, funding acquisition, writing–original draft, and writing–review and editing. In addition, NH contributed to supervision.

## Conflict of Interest

The authors declare that the research was conducted in the absence of any commercial or financial relationships that could be construed as a potential conflict of interest. The reviewer JS declared a shared affiliation with one of the authors CO to the handling editor at time of review.

## Publisher's Note

All claims expressed in this article are solely those of the authors and do not necessarily represent those of their affiliated organizations, or those of the publisher, the editors and the reviewers. Any product that may be evaluated in this article, or claim that may be made by its manufacturer, is not guaranteed or endorsed by the publisher.
